# Perioperative Liposomal Bupivacaine for Postoperative Pain Control in Cranial Neurosurgery: A Systematic Review

**DOI:** 10.1155/anrp/6822812

**Published:** 2026-11-03

**Authors:** Eyad Jamileh, Omar Farooq, Mohamed Xamza, Raees Samli, Abdullah Ahmed, Munir Ahmed

**Affiliations:** ^1^ School of Medicine Dentistry and Biomedical Sciences Queen’s University Belfast Belfast UK qub.ac.uk; ^2^ Department of Medicine Royal Blackburn Hospital East Lancashire Hospitals Trust Lancashire UK duhs.edu.pk; ^3^ Department of Medicine, Barking Havering and Redbridge University Hospitals NHS Trust London UK bhrhospitals.nhs.uk; ^4^ Department of Medicine Southend University Hospital Southend-on-Sea UK southend.nhs.uk; ^5^ Department of Physics Leyton Sixth Form College Leyton London UK

**Keywords:** craniotomy, liposomal bupivacaine, neurosurgery, postoperative pain, systematic review

## Abstract

**Background:**

Postcraniotomy pain is common and contributes to increased opioid use and delayed recovery. Liposomal bupivacaine (LB) is a long‐acting local anaesthetic that provides sustained analgesia for up to 96 h. Its role in cranial neurosurgery remains uncertain.

**Methods:**

A systematic review was conducted in accordance with PRISMA guidelines and registered prospectively on PROSPERO. MEDLINE, EMBASE, CINAHL, Web of Science, and CENTRAL were searched. Eligible studies assessed perioperative LB in elective cranial neurosurgical procedures and compared it with conventional local anaesthetic or no local infiltration. Primary outcomes were postoperative pain scores and opioid consumption expressed as morphine milligram equivalents. Secondary outcomes included length of stay, complications, and cost.

**Results:**

Four retrospective single‐centre studies involving 184 patients were included. One study examined adult aneurysm craniotomy, and three examined paediatric cranial vault remodelling or Chiari decompression. In the single adult aneurysm‐clipping cohort, mean pain scores, opioid consumption, and length of stay were lower with LB, but the between‐group differences were not statistically significant; reported total analgesic cost was significantly higher. In paediatric cohorts, some retrospective studies reported associations between LB use and lower opioid consumption, including during the first 24 h in one Chiari cohort and across the first three postoperative days in a cranial vault remodelling cohort. Effects on pain scores and length of stay were variable.

**Conclusion:**

Evidence for perioperative LB in cranial neurosurgery is sparse and of low to very low certainty. The single adult aneurysm‐clipping cohort found no statistically significant between‐group differences in pain scores, opioid consumption, or length of stay and reported higher total analgesic cost. Three small retrospective paediatric cohorts reported some associations with lower early opioid consumption, but findings were inconsistent across procedures and outcomes. Current evidence is insufficient to support routine use. Prospective, adequately powered randomised trials are required.

## 1. Introduction

Postcraniotomy pain is common and can substantially impact early recovery after intracranial surgery. Many patients experience moderate to severe pain within the first 24–48 h after intracranial surgery, which may contribute to increased opioid use, delayed mobilisation, and other complications [1–3]. Effective, nonopioid analgesic strategies are, therefore, essential, especially given the need to preserve neurological assessment and avoid side effects such as sedation, nausea, or respiratory compromise [4].

Traditionally, methods such as regional scalp‐nerve blocks (SNBs) or local anaesthetic infiltration (incisional or wound infiltration) have been used to reduce postcraniotomy pain and opioid consumption [5–7]. A recent network meta‐analysis of scalp blocks and infiltration techniques concluded that SNB using ropivacaine likely provides the greatest pain reduction and opioid‐sparing effect within the first 24 h postoperatively [8]. However, these conventional local anaesthetic techniques have limitations; the duration of analgesia is limited to the pharmacologic half‐life of the agent, often necessitating repeated dosing or reliance on systemic opioids as the block wears off [9].

In recent years, liposomal bupivacaine (LB), a long‐acting, extended‐release formulation of bupivacaine, has emerged as a promising analgesic for postoperative pain control in multiple surgical fields. Its encapsulation in lipid‐based vesicles enables sustained release over 72–96 h, covering the early postoperative pain period without the need for repeated dosing [10, 11].

LB has shown favourable results in nonneurosurgical settings, such as orthopaedic, general surgery, and maxillofacial surgery, where studies report reduced postoperative pain and opioid consumption [12–15]. However, its use in neurosurgery, specifically for cranial surgery, remains poorly studied. Clinicians, therefore, lack evidence‐based guidance for routine use in neurosurgical practice. Given the clinical importance of optimising postcranial surgery analgesia to reduce opioid requirements, improve recovery, and mitigate complications, this represents a significant gap in the literature. Therefore, a systematic review focused on perioperative LB in cranial neurosurgery is warranted.

This systematic review evaluates the available evidence regarding perioperative LB for postoperative pain control in cranial neurosurgery. It summarises reported postoperative pain, opioid consumption, recovery, complications, and resource‐use outcomes. It also identifies evidence gaps that require prospective clinical trials.

## 2. Methods

This systematic review was conducted in accordance with the Preferred Reporting Items for Systematic Reviews and Meta‐Analyses (PRISMA) guidelines [16]. The PRISMA checklist is provided in the supporting materials (S1).

### 2.1. Eligibility Criteria

Studies were eligible if they evaluated the effectiveness or safety of perioperative LB for postoperative analgesia in patients undergoing elective cranial neurosurgical procedures. Randomised controlled trials (RCTs), prospective cohort studies, and retrospective comparative studies were considered.

Included studies assessed LB administered via wound infiltration, incisional infiltration, or SCN and compared outcomes against standard analgesic regimens, conventional local anaesthetics, or no local infiltration. Eligible procedures included craniotomy for tumour resection, vascular neurosurgery, Chiari decompression, microvascular decompression, and other elective intracranial operations. Both adult and paediatric populations were included, with no restrictions on sex or surgical indication. Only English‐language publications were eligible.

Studies were excluded if they involved spinal surgery, trauma without craniotomy, peripheral nerve or craniofacial surgery, adenotonsillar or nonneurosurgical procedures, animal or cadaveric studies, case reports, and single‐arm studies without a comparator, narrative reviews, or editorials.

### 2.2. Outcomes

The primary outcomes were postoperative pain intensity measured using validated pain scales (including visual analogue scale or numeric rating scale) within 72 h following surgery, and total postoperative opioid consumption expressed as oral morphine equivalents.

Secondary outcomes included length of hospital stay, requirement for rescue analgesia, incidence of postoperative nausea and vomiting, wound‐related complications, neurological adverse events, overall postoperative complications, and hospital readmission, where reported.

### 2.3. Literature Search Strategy

A systematic literature search was independently performed by two reviewers across MEDLINE (via PubMed), EMBASE, CINAHL, Web of Science, and the Cochrane Central Register of Controlled Trials. The final search was conducted on the 24th of December 2025. Full search strategies are provided in the Supporting Materials (S2). Additional studies were identified through manual screening of reference lists and searches of ClinicalTrials.gov, the World Health Organisation International Clinical Trials Registry Platform, and the ISRCTN registry to identify ongoing or unpublished studies.

### 2.4. Study Selection

All retrieved records were screened independently by two reviewers based on titles and abstracts. Full‐text articles were reviewed for potentially eligible studies. Disagreements were resolved through discussion, with arbitration by a third reviewer when necessary.

### 2.5. Data Extraction

Data were extracted independently by two reviewers using a standardised and pilot‐tested extraction form. Extracted variables included study characteristics (author, year, country, design, and sample size), patient demographics, surgical procedure, method and dose of LB administration, comparator details, and all reported primary and secondary outcomes. Any discrepancies were resolved by consensus.

### 2.6. Risk of Bias and Quality Assessment

Because all included studies were nonrandomised retrospective comparative studies, risk of bias was assessed using the Newcastle–Ottawa Scale. This tool was selected because it is commonly used to assess the methodological quality of observational cohort studies, including the selection of study groups, comparability of cohorts, and ascertainment of outcomes. Quality assessment was conducted independently by two reviewers, with disagreements resolved by consensus. For studies only available as conference abstracts, methodological assessment was restricted to information explicitly reported in the abstract. Newcastle–Ottawa Scale items that could not be verified from the available report were not assumed to have been fulfilled.

The certainty of evidence for key outcomes was assessed using the GRADE framework. Outcomes assessed included postoperative opioid consumption, postoperative pain scores, length of stay, complications, and cost. Certainty of evidence was rated as high, moderate, low, or very low based on risk of bias, inconsistency, indirectness, imprecision, and publication bias.

### 2.7. Data Synthesis

Due to substantial clinical and methodological heterogeneity among the included studies, quantitative meta‐analysis was not performed. Studies differed in patient population, including adult and paediatric cohorts; surgical indication, including aneurysm clipping, Chiari decompression, and cranial vault remodelling; method of LB administration; comparator group; perioperative analgesic regimen; pain assessment tool; opioid reporting method; and timing of outcome assessment. Given these differences, statistical pooling was considered inappropriate and potentially misleading. Findings were, therefore, synthesised narratively and grouped according to population, procedure type, and outcome domain.

Publication bias was not formally assessed because only four studies were included and quantitative meta‐analysis was not performed. Funnel plot assessment and statistical tests for small‐study effects were, therefore, not appropriate.

## 3. Results

### 3.1. Literature Search

The database search identified 214 records. After removal of 16 duplicates, 198 records remained for title and abstract screening. Of these, 193 records were excluded, including reviews or case reports (*n* = 43), studies with a different comparison (*n* = 48), unrelated topics (*n* = 50), abstract‐only records (*n* = 2), and studies involving an ineligible population (*n* = 50). Five full‐text reports were assessed for eligibility. Following full‐text review, one report was excluded because of an ineligible population. Four studies met the eligibility criteria and were included in the final narrative synthesis (Figure 1).

**FIGURE 1:**
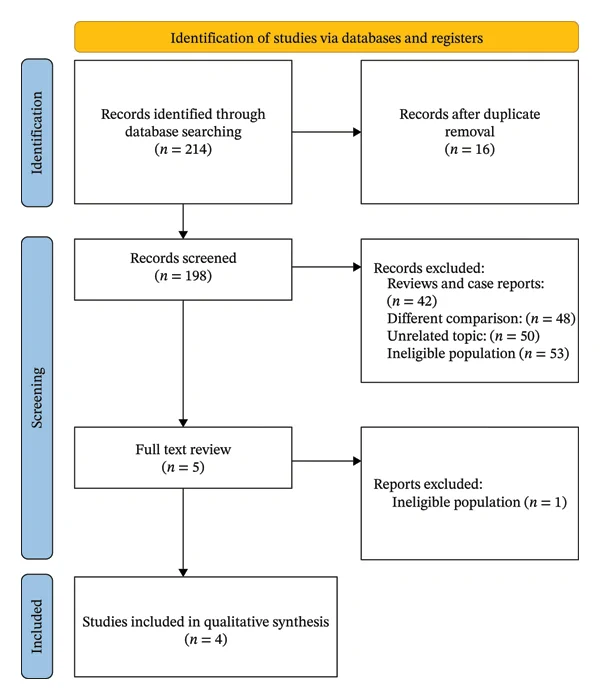
PRISMA flowchart.

### 3.2. Study Characteristics

Four retrospective single‐centre studies were included [17–20]. One study evaluated an adult cranial neurosurgical population, while three studies evaluated paediatric cranial neurosurgical populations. Because adult and paediatric cranial neurosurgery differ substantially in surgical indication, analgesic requirements, perioperative care pathways, and pain assessment methods, findings are presented separately where appropriate.

The adult cohort was reported by Laing et al. and involved adults undergoing elective clipping of unruptured anterior circulation cerebral aneurysms via pterional or lateral supraorbital craniotomy, comparing intrawound LB with no local anaesthetic [17].

Three paediatric cohorts were identified [18–20]. Edala et al., a conference abstract representing grey literature, evaluated paediatric patients undergoing cranial vault remodelling for craniofacial reconstruction, comparing a preincision scalp block with LB versus conventional bupivacaine [18]. LoPresti et al. studied paediatric patients aged 0–18 years undergoing elective posterior fossa decompression with C1 laminectomy for Chiari malformation, comparing intrawound LB with no local infiltration [19]. Lu et al. assessed paediatric patients undergoing Chiari I malformation posterior fossa decompression, comparing a single intraoperative dose of LB with no LB [20]. Table 1 summarises the study characteristics.

**TABLE 1:** Baseline study characteristics.

Author (Year)	Study design	Centre type	Mean age ± SD	Sex (male: female)	LB group (*n*)	Control group (*n*)
Laing et al. (2025)	Retrospective	Single centre	LB 60.41 ± 4.68, control 59.43 ± 4.36	13:66	44	36
Edala et al. (2022)	Retrospective	Single centre	NR	NR	29	29
LoPresti et al. (2021)	Retrospective	Single centre	LB 6.1 ± 2.2 (3.5–10.0), control 10.1 ± 5.0 (2.3–18.6)	11:19	6	24
Lu et al. (2020)	Retrospective	Single centre	15.9 (SD NR)	5:13	9	9

Abbreviation: LB, liposomal bupivacaine.

### 3.3. Opioid Usage

#### 3.3.1. Adult Studies

In the adult aneurysm craniotomy cohort reported by Laing et al. 2025, mean opioid consumption measured in morphine milligram equivalents was lower in the LB group than in controls, 55.95 ± 16.29 versus 77.76 ± 26.97 MME; however, this difference was not statistically significant, *p* = 0.3046 [17].

#### 3.3.2. Paediatric Studies

In the paediatric cranial vault remodelling study by Edala et al., the LB group required fewer opioids on each of the first three postoperative days than the conventional bupivacaine group, and this reduction was statistically significant [18]. In the LoPresti et al. Chiari cohort, inpatient opioid consumption was lower in the LB group, 3.6 ± 5.9 versus 12.2 ± 13.0 MME, although this difference was not statistically significant [19]. In the Lu et al. 2020 Chiari I cohort, opioid use within the first 24 h was significantly lower with LB, 17.5 versus 47.9 MME, *p* = 0.03; however, opioid use from 24 h to discharge did not differ significantly, *p* = 0.51 [20].

### 3.4. Postoperative Pain Scores

#### 3.4.1. Adult Studies

In the adult cohort by Laing et al., pain scores measured on a 10‐point Numerical Rating Scale were 3.55 ± 0.64 in the LB group and 4.05 ± 0.75 in controls; this difference was not statistically significant, *p* = 0.1611 [17].

#### 3.4.2. Paediatric Studies

In the Edala et al. study, postoperative pain scores were not reported [18]. In the LoPresti et al. cohort, pain was assessed using the VAS and FLACC scales, with no significant difference in subjective pain control or maximum VAS or FLACC scores between groups [19]. In the Lu et al. study, mean pain scores in the first 24 postoperative hours were significantly lower with LB, 3.6 versus 5.5 on the 10‐point Visual Analogue Scale, *p* = 0.04; from 24 h to discharge, the difference was not statistically significant, *p* = 0.09 [20].

### 3.5. Length of Stay

#### 3.5.1. Adult Studies

In the adult cohort reported by Laing et al., hospital length of stay was shorter in the LB group than in the control group, 3.16 ± 0.65 days versus 3.66 ± 0.88 days, respectively [17]. However, this difference was not statistically significant, *p* = 0.0778.

#### 3.5.2. Paediatric Studies

In the paediatric cranial vault remodelling cohort reported by Edala et al., patients receiving LB were discharged a mean of 1.2 days earlier than controls [18]. This difference was statistically significant, *p* = 0.0108. In the paediatric Chiari cohort reported by LoPresti et al., length of stay was 2.5 ± 1.0 days in the LB group and 3.4 ± 1.2 days in the control group [19]. This difference was not statistically significant. In the Lu et al. paediatric Chiari I cohort, the overall mean length of stay was 2.7 days; however, group‐specific comparisons were not reported [20].

#### 3.5.3. Cost

Only Laing et al. reported a cost outcome [17]. The reported measure was total analgesic cost, which was significantly higher in the LB group than in controls (647.84 ± 122.50 versus 284.77 ± 113.44 dollars, *p* < 0.0001). The study did not report total hospitalisation costs or undertake a formal cost‐effectiveness analysis.

#### 3.5.4. Methodological Quality and Risk of Bias Assessment

All four observational studies were evaluated using the Newcastle–Ottawa Scale, summarised in Table 2. According to the Agency for Healthcare Research and Quality standards, all the studies were rated to be of fair quality. Using the GRADE framework, the certainty of evidence was judged to be low to very low across outcomes. Evidence for opioid consumption and postoperative pain scores was rated as low because findings were derived from retrospective observational studies with small sample sizes and inconsistent outcome measurement. Evidence for length of stay, complications, and cost was rated as very low because of imprecision, limited reporting, and substantial clinical heterogeneity. These assessments are summarised in Table 3.

**TABLE 2:** Newcastle–Ottawa Scale to assess the quality of nonrandomised studies.

Author (year)	Selection (out of 4 stars)	Comparability (out of 2 stars)	Outcome/exposure (out of 3 stars)	Quality assessment based on the agency for healthcare research and quality (AHRQ)
Laing et al. (2025)	^∗∗∗^	^∗^	^∗∗^	Fair
Edala et al. (2022)	^∗∗^	^∗^	^∗∗^	Fair
LoPresti et al. (2021)	^∗∗^	^∗^	^∗∗∗^	Fair
Lu et al. (2020)	^∗∗∗^	^∗^	^∗∗^	Fair

^∗^ = 1 star.

^∗∗^ = 2 stars.

^∗∗∗^ = 3 stars.

^∗∗∗∗^ = 4 stars.

**TABLE 3:** GRADE summary of findings.

Outcome	No. of studies	Main finding	Certainty of evidence	Main reasons for rating
Opioid consumption	4	Some paediatric studies reported reduced opioid consumption, particularly within the first 24 h; the adult study showed no statistically significant reduction	Low	Retrospective designs, small sample sizes, heterogeneity, inconsistent opioid reporting
Postoperative pain scores	3	Findings were variable; one paediatric study reported significantly lower pain scores in the first 24 h, while other studies showed no significant difference	Low	Different pain scales, inconsistent time points, imprecision
Length of hospital stay	4	One paediatric cranial vault remodelling study reported shorter length of stay; other studies did not show consistent benefit	Very low	Confounding, heterogeneous procedures, small samples, incomplete reporting
Complications/safety	4	Adverse‐event reporting was insufficient to permit reliable conclusions regarding comparative safety	Very low	Limited adverse‐event reporting, small sample sizes, short follow‐up
Cost	1	The single adult aneurysm‐clipping cohort reported significantly higher total analgesic cost with liposomal bupivacaine; total hospital costs and formal cost‐effectiveness were not assessed [17].	Very low	Single study, no formal cost‐effectiveness analysis

## 4. Discussion

This systematic review demonstrates that the evidence base for perioperative LB in cranial neurosurgery remains sparse. Only four retrospective, single‐centre comparative studies were identified, comprising one adult aneurysm‐clipping cohort and three paediatric cohorts involving different procedures. The studies were small, clinically heterogeneous, and used inconsistent analgesic protocols and outcome measures. Accordingly, the principal finding of this review is not evidence of a consistent treatment effect, but rather the absence of sufficiently robust evidence to determine the clinical effectiveness of LB in cranial neurosurgery.

Evidence in adults was limited to a single retrospective cohort of patients undergoing elective clipping of unruptured cerebral aneurysms. Although mean pain scores, opioid consumption, and length of stay were numerically lower in patients receiving LB, none of these between‐group differences reached statistical significance. The study also reported significantly higher total analgesic cost with LB. Given the single‐centre retrospective design and limited sample size, these findings cannot be generalised to adult cranial neurosurgery more broadly and do not establish either benefit or absence of benefit. In paediatric cranial neurosurgery, evidence was derived from three retrospective single‐centre studies involving cranial vault remodelling and Chiari decompression. Some studies reported associations with lower short‐term opioid consumption, particularly within the first 24 h, and one cranial vault remodelling study reported a shorter length of stay. However, findings were inconsistent across outcomes, and the included studies were limited by small sample sizes, retrospective designs, and heterogeneous analgesic protocols. These findings are suggestive of a possible association in selected paediatric procedures but are insufficient to establish a treatment effect or support routine use across paediatric cranial neurosurgery. The observed pattern of benefit in selected paediatric populations aligns with the broader surgical literature, where LB shows variable efficacy across specialties. In orthopaedic, general, and maxillofacial surgery, opioid sparing effects are inconsistent and often modest [21–24]. The limited cranial neurosurgical evidence appears broadly consistent with this variable pattern. The available retrospective data raise the possibility that LB may warrant further study in selected paediatric procedures, including posterior fossa decompression and complex craniofacial reconstruction. The timing of benefit appears limited to the early postoperative window, particularly within the early postoperative period [11, 22]. However, this interpretation remains uncertain because the available cranial neurosurgical evidence is limited to small retrospective studies.

Only one study reported cost data: the adult aneurysm‐clipping cohort [17]. That study reported significantly higher total analgesic cost with LB, while differences in pain scores, opioid consumption, and length of stay were not statistically significant [17]. However, the authors did not evaluate total hospitalisation costs or formal cost‐effectiveness outcomes [17]. Consequently, the available evidence is insufficient to conclude the cost‐effectiveness of LB in cranial neurosurgery. Adverse‐event reporting was limited and inconsistent across the included studies. The small sample sizes and retrospective designs preclude reliable conclusions regarding the safety of LB in cranial neurosurgery, particularly for uncommon adverse events. Accordingly, this review cannot establish the comparative safety of LB in this setting.

### 4.1. Limitations

The conclusions of this systematic review are limited by the overall quality of the available evidence. All included studies were retrospective and single‐centre in design, introducing a high risk of selection bias, confounding, and incomplete outcome capture. No RCTs with published outcome data were available. Sample sizes were small, particularly in the paediatric Chiari cohorts, limiting statistical power and increasing the risk of type II error. There was substantial clinical heterogeneity in patient age, procedure type, method of LB administration, and comparator analgesic techniques. Outcome reporting was inconsistent, with incomplete opioid quantification, absent variance measures, variable pain assessment tools, and limited cost data. These limitations precluded formal quantitative synthesis and restricted analysis to a qualitative narrative review, thereby limiting the strength of causal inference. Publication bias could not be formally assessed because of the limited number of included studies and the absence of quantitative meta‐analysis.

This review also highlights several critical evidence gaps. Future trials should focus on randomised, adequately powered, procedure‐specific studies with standardised reporting of pain scores, opioid consumption in morphine milligram equivalents, length of stay, and formal cost‐effectiveness analysis. Studies should compare LB against contemporary multimodal analgesic protocols rather than historical controls. Dose optimisation, method of delivery, and patient selection criteria must be formally evaluated. Longitudinal outcomes, including mobilisation time, functional recovery, and patient‐reported satisfaction, should be incorporated to determine true clinical value.

## 5. Conclusion

Evidence regarding perioperative LB in cranial neurosurgery remains sparse, heterogeneous, and of low to very low certainty. The single adult aneurysm‐clipping cohort did not demonstrate statistically significant between‐group differences in pain scores, opioid consumption, or length of stay and reported higher total analgesic cost, but these findings cannot be generalised to adult cranial neurosurgery more broadly. Three small retrospective paediatric studies reported some associations with lower early opioid consumption, although findings were inconsistent across procedures and outcomes. The available evidence is insufficient to establish clinical effectiveness or comparative safety, or to support routine use. Adequately powered prospective randomised trials with standardised outcome reporting are required.

## Author Contributions

Eyad Jamileh collected and analysed data, contributed to drafting, and revised the article. Mohamed Xamza, Raees Samli, and Abdullah Ahmed collected and analysed data and contributed to drafting. Munir Ahmed and Omar Farooq critically revised the manuscript and contributed to final approval.

## Funding

The authors received no financial support for the research, authorship, and/or publication of this article.

## Disclosure

The abstract of this manuscript has been presented at the Society of British Neurological Surgeons Spring Meeting in April 2026, in Harrogate, United Kingdom.

## Ethics Statement

This systematic review was registered in the PROSPERO International Register of Systematic Reviews. Registration number: CRD420251248772 [25]. The protocol is available from https://www.crd.york.ac.uk/PROSPERO/view/CRD420251248772. No amendments were made to the protocol.

## Consent

The authors have nothing to report.

## Conflicts of Interest

The authors declare no conflicts of interest.

## Supporting Information

Additional supporting information can be found online in the Supporting Information section.

## Supporting information


**Supporting Information 1** Supporting 1 (S1): PRISMA checklist for Systematic Reviews and Meta‐Analyses.

**Supporting Information 2** Supporting 2 (S2): Complete search strategy.

## Data Availability

Data are available upon request from authors.
