# Supplementary material for: Perioperative Liposomal Bupivacaine for Postoperative Pain Control in Cranial Neurosurgery: A Systematic Review
Source: Anesthesiol Res Pract. 2026 Nov 3;2026:6822812. doi: 10.1155/anrp/6822812 (PMC13728994; doi:10.1155/anrp/6822812)
Supplement: Supplementary file 1 [file ANRP-2026-6822812-s001.docx]

# Supplementary Material (S2) Search Strategy

A comprehensive literature search was conducted to identify studies evaluating the use of perioperative liposomal bupivacaine for postoperative pain control in cranial neurosurgery. The following electronic databases were searched: MEDLINE (via PubMed), EMBASE, CINAHL, Web of Science, and the Cochrane Central Register of Controlled Trials (CENTRAL). The final search was conducted on the prespecified search date. No restrictions were placed on publication date. Only studies published in English were included.

## MEDLINE (PubMed)

(“liposomal bupivacaine” OR “bupivacaine liposome” OR Exparel)
AND
(craniotomy OR “cranial surgery” OR neurosurgery OR intracranial)
AND
(“postoperative pain” OR analgesia OR pain OR opioid OR “opioid consumption”)

## EMBASE

(liposomal bupivacaine OR bupivacaine liposome OR Exparel)
AND
(craniotomy OR cranial surgery OR neurosurgery OR intracranial)
AND
(postoperative pain OR analgesia OR pain OR opioid OR opioid consumption)

## CINAHL

(liposomal bupivacaine OR bupivacaine liposome OR Exparel)
AND
(craniotomy OR cranial surgery OR neurosurgery OR intracranial)
AND
(postoperative pain OR analgesia OR pain OR opioid)

## Web of Science

TS=(liposomal bupivacaine OR bupivacaine liposome OR Exparel)
AND
TS=(craniotomy OR cranial surgery OR neurosurgery OR intracranial)
AND
TS=(postoperative pain OR analgesia OR pain OR opioid)

## CENTRAL

(liposomal bupivacaine OR bupivacaine liposome OR Exparel)
AND
(craniotomy OR cranial surgery OR neurosurgery OR intracranial)
AND
(postoperative pain OR analgesia OR pain OR opioid)

Reference lists of all included studies and relevant review articles were screened manually to identify additional eligible studies. Grey literature was assessed by searching ClinicalTrials.gov and the WHO International Clinical Trials Registry Platform for ongoing or unpublished studies.
